# The influence of psychological network on the willingness to communicate in a second language

**DOI:** 10.1371/journal.pone.0256644

**Published:** 2021-09-17

**Authors:** Takehiko Ito

**Affiliations:** Department of Information Networking for Innovation and Design, Toyo University, Tokyo, Japan; Kyoto University, JAPAN

## Abstract

This study investigated the effect of the psychological network on the willingness to communicate in English among Japanese people. Previous studies have shown that psychological factors affect the willingness to communicate in English for Japanese people. However, the network structure of psychological factors and their effects have not been revealed yet. The present study conducted a network analysis with 644 Japanese people. Consequently, the edge between perceived communication competence and the willingness to communicate in the first or second language was very strong. Node centrality strength showed that these factors were central in the network structure. The results of the network analysis show the effect of psychological networks on the willingness to communicate in a second language, which will be beneficial for language education.

## Introduction

Many studies have addressed the psychological processes that shape communication attitudes toward second languages; they first focused on Canadians [1, 2]. Several studies have also been performed among Asian individuals, such as Chinese [3], Iranians [4], Japanese [5, 6], and Malaysians [7]. Researchers have proposed the willingness to communicate (WTC) as a positive attitude toward language communication [2]. The WTC in a second language is defined as readiness to enter into a discussion voluntarily at a specific time with one or more people using a second language [2]. Positive correlations between the WTC and the frequency of communication in a second language have been reported [1, 6].

However, previous studies have not examined the network structure of psychological factors and their effect on the WTC. The interactions between the factors and the extent of influence that each factor holds have not been explored. This study conducted a network analysis to examine the network structure of psychological factors. The position of the nodes in the network is based on an algorithm that makes strongly correlated factors cluster in the middle, while factors with weaker connections to other factors go to the periphery [8].

We could determine the effect of each psychological factor on the WTC and the correlations between the factors with a regression analysis. However, the regression analysis does not identify the psychological factor that serves as the center (hub) between factors in relation to the WTC. If we could identify the centrality, we would better understand the factor that is essential for enhancing the WTC and the influence of each psychological factor [9]. Using this analysis, the effects can be seen visually; therefore, it is useful for intervention studies [9].

Several psychological factors related to the WTC in a second language were used based on the previous studies to reveal the psychological network structure to predict the WTC in English for Japanese people. First, perceived communication competence in English was used because MacIntyre et al. [2] insisted that WTC in a second language has a stronger positive correlation with confidence in a second language than actual competence in the second language. Second, communication anxiety in English was used because MacIntyre and Charos [1] showed that second language anxiety was negatively correlated with the WTC in the second language. Third, the willingness to communicate in Japanese and perceived communication competence in Japanese were used because the WTC in a second language was positively related to the WTC in the first language [10]. Fourth, general trust was used because Ito [5] performed a regression analysis that found that general trust positively influenced the WTC in English for Japanese people. Fifth, the Big Five personality traits were used because the WTC in a second language was positively related to some of the Big Five personality traits [1]. In fact, the Big Five personality traits indirectly influenced the WTC in a second language via perceived competence and anxiety. Therefore, to examine the relationship between perceived communication competence, communication anxiety, and WTC in English, these personality factors were important as the control variables.

General trust is a personality trait that indicates how much individuals trust others in general [11]. People higher in general trust are more likely to obtain social benefits than those who are lower because the former work harder to maximize profitable relations by interacting with larger networks of people [12]. Based on this perspective, general trust is assumed to be positively associated with WTC in a second language because it is a personality trait that builds one’s social network [5].

As for the Big Five personality traits, there are no consistent results regarding which aspects relate to WTC. MacIntyre and Charos [1] showed that openness influenced WTC in a second language via perceived competence, whereas extraversion influenced WTC via anxiety, but agreeableness was directly related to WTC in a second language. According to Oz [13], there were positive correlations between openness to experience, extraversion, agreeableness, and language learners’ English WTC. Furthermore, MacIntyre et al. [14] outlined that introversion/extroversion and emotional stability were related to WTC through perceived language competence and communication apprehension. Additionally, Karadag and Kaya [15] showed that there were positive correlations between WTC and extraversion, openness to experience, and conscientiousness.

## Methods

### Participants

The present study analyzed the data collected by Ito [9]. The participants of the online social survey were 644 Japanese people living in Tokyo (310 men, 334 women; mean age = 39.41 years, *SD* = 11.20). The age criteria for participant recruitment was from twenties to fifties, who have registered the survey company “Just System”. We stopped recruiting when the total number of participants was over 150 for each age group. The demographic details were shown in Table 1. According to Epskamp [16], for the psychological network analysis, the correlation between true and estimated edge weights in the network is good for sample sizes greater than 250. Therefore, the number of participants being more than 600 was enough. Furthermore, to have an approximately equal number of people from each generation, there were four age groups including about 150 people each selected.

**Table 1:** The number of participants for each category.

Age Sex	Men	Women	Total
**20 ~ 29**	46	122	168
**30 ~ 39**	62	103	165
**40 ~ 49**	103	61	164
**50 ~ 59**	99	48	147
**Total**	310	334	644

In this study, the “chance of communicating with English speakers” was measured. The participants answered the following question, “In daily life, how often do you talk with English speakers?” Answers were 1  =  *Not at all*, 2  =  *Once a month*, 3  =  *Once a week*, 4  =  *Three times a week*, and 5  =  *Every day*. Th results showed that 277 participants had no opportunity to talk with English speakers in their daily lives at all. In Japan, people study English since elementary or junior high school, and they communicate in English at least in classroom. Therefore, the participants’ familiarity with the target language would be retained in the present study even if 277 participants did not speak in English in their daily lives at all.

As for their English learning experience, the participants were asked whether they had any opportunities to stay in foreign countries, and 353 of them answered with yes. As for their objective English proficiency, EIKEN (Test in Practical English Proficiency) was conducted. A total of 339 participants answered their grade (Grade 1 is top: 25 participants for Grade 1, 38 for Grade Pre-1, 92 for Grade 2, 54 for Grade Pre-2, 86 for Grade 3, 36 for Grade 4, and 8 for Grade 5).

### Procedure

Data for the present study were collected from 4 through 11 February 2020. The survey was conducted online using a web-based survey tool called Fastask. The survey company sent emails with links to the online system of questionnaires with scales assessing the psychological factors and the WTC in English to the participants. The participants could access the links on their personal computers at their convenience. The first page of the questionnaire contained information written in Japanese stating that the participation was voluntary and anonymous. The participants provided informed consent to participate. The participants received compensation after the survey was complete. The Research Committee of the Center for English as a Lingua Franca at Tamagawa University approved this study. All methods were performed in accordance with the relevant guidelines and regulations.

### Questionnaires

#### Willingness to communicate in English

The WTC scale was published by McCroskey [17], and the Japanese version [6] was used in the present study. The participants indicated the extent of their willingness to perform what each item described in English. The scale contained four communication contexts (talking in dyads, small groups, large meetings, and in front of an audience) with three type of receivers: strangers, acquaintances, and friends. Twelve items were used for the WTC in English (S1 Data; *α* = .97, acquaintances: e.g., “Talking in a large meeting of acquaintances”; strangers: “Talking with a stranger”; friends: “Talking with a small group of friends.”) The response options for all the statements ranged from 1 (*not at all*) to 5 (*very much*).

#### Perceived communication competence in English

This scale [1] measures the self-perception of communication competence. The participants indicated their self-assessed competency in English communication in the same contexts and with the same receivers as in the WTC scale in English (12 items, *α* = .95). The Japanese version was used [6]. The response options for all the statements ranged from 1 (*no competency*) to 5 (*enough competency*).

#### Communication anxiety in English

The item “I feel anxious when I speak English” was used to measure communication anxiety in English. The response options for this item ranged from 1 (*strongly disagree*) to 5 (*strongly agree*). Previous study [6] used the same 12 items for WTC, perceived communication competence, and communication anxiety. It was possible for participants to answer all three questionnaires the same way without paying attention to the instructions. Therefore, the items of communication anxiety were changed. In the present data, the negative correlation between perceived communication competence and communication anxiety was shown (*r* = -.35, *p* < .01), which is the pattern present in the previous study [6], and the reliability of the scale was retained.

#### Willingness to communicate in Japanese

The participants indicated the extent of their willingness to perform what each item described in Japanese, and each item was the same as the WTC scale in English (12 items, *α* = .95). The response options for all the statements ranged from 1 (*not at all*) to 5 (*very much*).

#### Perceived communication competence in Japanese

The participants indicated their self-assessed competency in Japanese communication in the same contexts and with the same receivers as in the WTC scale in English (12 items, *α* = .97). The response options for all the statements ranged from 1 (*no competency*) to 5 (*enough competency*).

#### General trust

Six items were used to assess participants’ general trust (S2 Data; *α* = .89). The scale was published by Yamagishi and Yamagishi [18], and the Japanese version was used [11]. The examples of the items are as follows: “Most people are trustworthy” and “Most people are basically good and kind.” The response options for all the statements ranged from 1 (*strongly disagree*) to 5 (*strongly agree*).

#### Big five personality traits

We used the Japanese version [19] of the short form of the Big-Five scale, the “Ten-Item Personality Inventory” [20]. Its reliability and validity have been verified [21]. Participants indicated the extent of the perceived personality traits by responding to the 10 items. This scale comprised five personality factors, namely, extraversion: “Extraverted, enthusiastic” and “Reserved, quiet” (reverse-scored; *r* = .19, *p* < .01); conscientiousness: “Dependable, self-disciplined” and “Disorganized, careless” (reverse-scored; *r* = .12, *p* < .01); neuroticism: “Anxious, easily upset” (reverse-scored) and “Calm, emotionally stable” (*r* = .18, *p* < .01); openness: “Open to new experiences, complex” and “Conventional, uncreative” (reverse-scored; *r* = .15, *p* < .01); agreeableness: “Critical, quarrelsome” (reverse-scored) and “Sympathetic, warm” (*r* = .00, *n*.*s*.). The response options for all the statements ranged from 1 (*strongly disagree*) to 5 (*strongly agree*).

## Results

The mean and standard deviation of WTC (*M* = 2.52, *SD* = 1.14), competence (*M* = 2.52, *SD* = 1.18), and anxiety (*M* = 3.66, *SD* = 1.38) were analyzed. As a whole, there were no floor effects. Furthermore, the mean and standard deviation of WTC (*M* = 2.46, *SD* = 1.23), competence (*M* = 2.60, *SD* = 1.29), and anxiety (*M* = 3.65, *SD* = 1.50) for participants in their 50s were analyzed, and floor effects were not confirmed.

Before conducting the network analysis, we performed hierarchical multiple regression analysis to examine the effects of psychological factors on the WTC in English (Table 2). After controlling communication variables in Japanese and general personality traits, perceived communication competence in English had a strong positive effect on WTC in English (β = .61, *p* < .01), and communication anxiety in English had a negative effect (β = -.12, *p* < .01). The delta R2 in Step 2 was .34 (*p* < .01). Therefore, consistent with previous studies [22], the effect of perceived communication competence in English was the strongest for the WTC in English.

**Table 2:** Results of hierarchical multiple regression analysis of psychological factors on the WTC in English.

Variable	Step 1	Step 2
	β	β
Sex	.00	.01
Age	-.13**	-.07*
WTC in Japanese	.17**	.17**
Perceived communication competence in Japanese	.22**	.05
General trust	.19**	.11**
Extraversion	.02	-.03
Conscientiousness	.10*	.04
Neuroticism	.13**	.04
Openness	.01	-.04
Agreeableness	-.14**	-.04
Perceived communication competence in English		.61**
Communication anxiety in English		-.12**
*R* ^2^	.30**	.64**
Δ*R*^2^		.34**

**p< .01

*p< .05

^+^*p*< .10.

To examine the network structure of psychological factors and their effect on the WTC in English, we used LASSO (least absolute shrinkage and selection operator) regularization with EBIC (minimizing the extended Bayesian information criterion) model selection [16]. The LASSO shrinks partial correlation coefficients for estimating a network structure whose edges show the value of association between nodes, after controlling for the influence of all other nodes in the network and sets small coefficients to zero [16]. EBIC was used to identify the true network structure [23, 24]. Therefore, LASSO regularization with EBIC does not estimate edges that are not in the true network, it estimates edges that are in the true network based on the true network structure and sample size [16].

**Fig 1:**
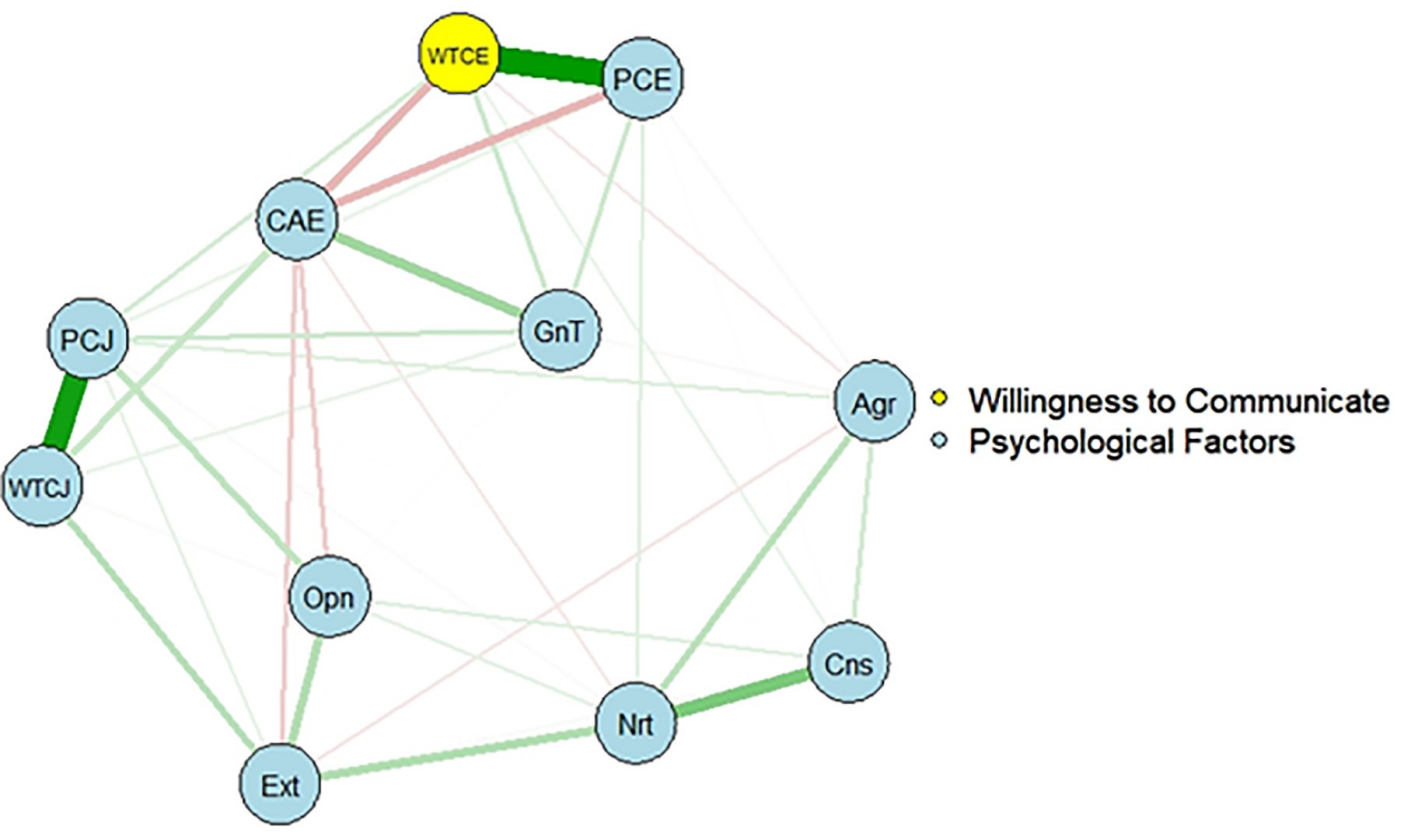
The network structure of psychological factors and the WTC in English. Nodes represent observed variables, and links represent partial correlations between two factors, after controlling for all other factors. Green link means positive correlations and red link means negative correlations. WTCE = Willingness to Communicate in English; PCE = Perceived communication competence in English; CAE = Communication anxiety in English; WTCJ = Willingness to Communicate in Japanese; PCJ = Perceived communication competence in Japanese; GnT = General Trust; Ext = Extraversion; Cns = Conscientiousness; Nrt = Neuroticism; Opn = Openness; Agr = Agreeableness.

The R package *qgraph* [25] and *glasso* [26] were used to estimate a partial correlation network using EBIC selection (Fig 1). The edge between the perceived communication competence in English and the WTC in English was very thick, which indicates a strong positive connection. The tendency was the same as the edge between the perceived communication competence in Japanese and the WTC in Japanese. General trust also had a positive connection with the WTC in English. While many factors had positive connections with the WTC in English, communication anxiety in English had a negative connection. The Big Five personality traits made a cluster that had weak impacts on the WTC in English. Even if age and sex were controlled in the network, the network structure was not changed, meaning that the components of each cluster were the same (S1 Fig).

The importance of each node in the network is indicated by node centrality. Node strength sums up the strength of all connected edges to a node. The network is the partial correlation coefficient, and the node strength shows the sum of the absolute partial correlation coefficients between the node and all other nodes [16]. Closeness indicates the inverse of the sum of all the shortest paths between the node and all other nodes in the network [16]. Betweenness refers to the number of shortest paths between two nodes passing through the node; the higher the betweenness, the more important the node is in connecting other nodes [16].

**Fig 2:**
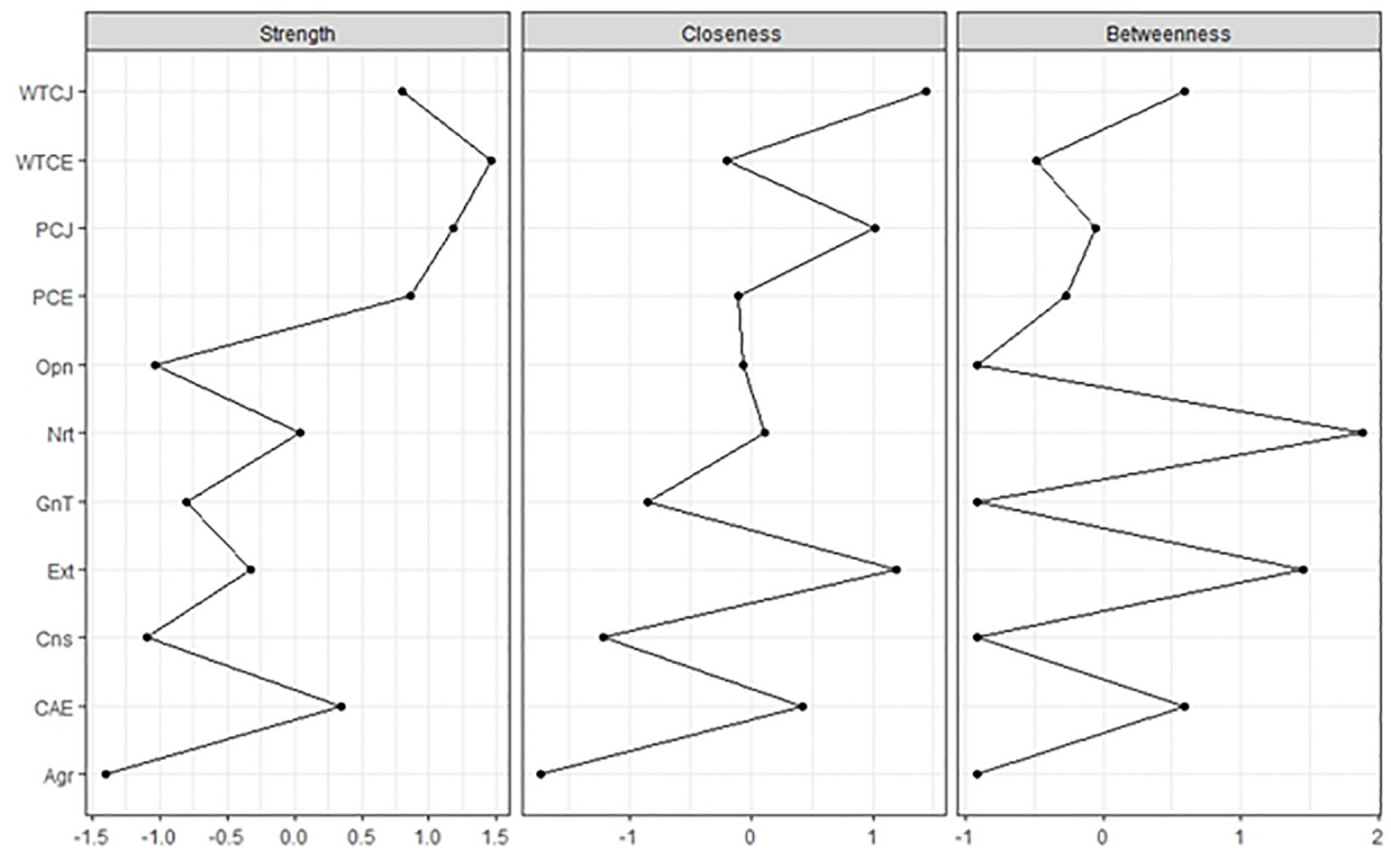
Centrality indices are shown as standardized z-scores. The abbreviations are the same as Fig 1.

Function centrality plot was used to estimate the centrality of the partial correlation network using EBIC selection [23] (Fig 2). For strength, the WTC in English had the highest score. The WTC in Japanese, WTC in English, perceived communication competence in Japanese, and perceived communication competence in English had scores of around 1.0. Even if age and sex were controlled in the network, the WTC in English had the highest score (S2 Fig). For closeness, the WTC in Japanese had the highest score. The WTC in Japanese, perceived communication competence in Japanese, and extraversion had scores around 1.0. For betweenness, neuroticism had the highest score. Neuroticism and extraversion had scores over 1.0.

Bootstrapping methods were used to overcome replication concerns. The *bootnet* package can estimate different network models and assess the accuracy of the estimated network structure [16]. The package includes bootstrapping methods, *the CS*-coefficient, and bootstrapped difference tests.

**Fig 3:**
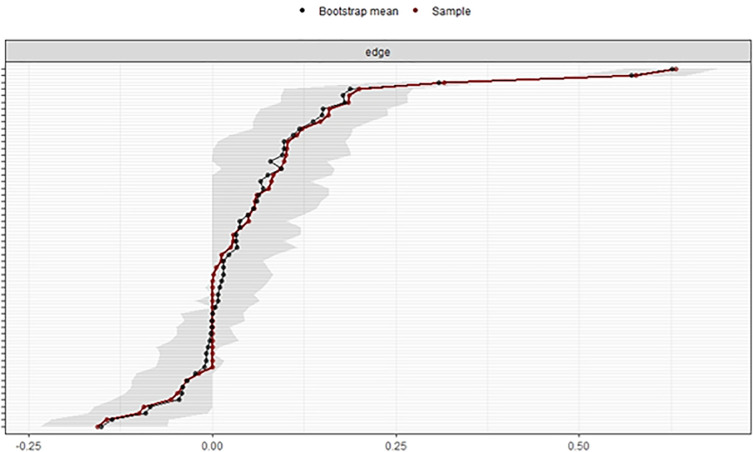
Bootstrapped confidence intervals of estimated edge weights for the estimated network. The red line shows the sample values, and the gray area shows the bootstrapped CIs. Each horizontal line represents one edge of the network. The top is the highest edge weight, and the bottom is the lowest edge weight. Y-axis labels were removed to avoid cluttering.

Fig 3 depicts bootstrapped CIs around the estimated edge weights, indicating that many edge weights probably do not differ significantly from one another [16]. The edges between perceived communication competence in English and the WTC in English and between perceived communication competence in Japanese and the WTC in Japanese are reliable because their bootstrapped CIs do not overlap with the bootstrapped CIs of any other edges. Non-overlapping CIs indicate that the two statistics differ significantly at the given significance level [16]. In the *bootnet* function, the *nBoots* (n = 2500, this time) argument was used to achieve smoother plots [16]. The *nCores* (n = 8, this time) argument was used to speed up bootstrapping and use multiple computer cores. The *plot* function was used to plot bootstrapped CIs for estimates.

**Fig 4:**
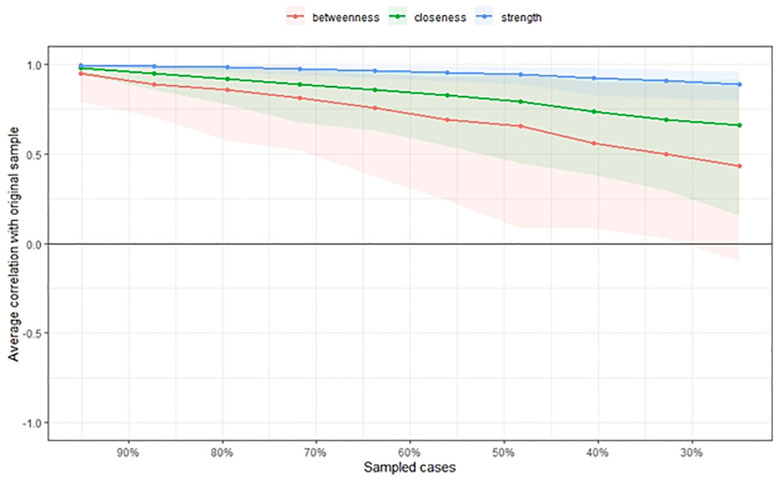
Average correlations between the centrality indices of networks sampled with participants dropped and the original participants. Colored lines depict the means, and areas depict the range from the 2.5th quantile to the 97.5th quantile.

Next, we examined the stability of the centrality indices of the estimated network models based on subsets of the data. Case-dropping bootstrapping by the *corStability* function was used. Fig 4 shows the resulting plot: the stability of closeness and betweenness decreased steeply, while the stability of node strength improved. The *CS coefficient* calculates the maximum proportion of cases that can be dropped to retain a correlation with the original centrality of higher than (by default) 0.7 [16]. The *CS coefficient* of betweenness was not good (*CS*(cor = 0.7) = 0.13) as did closeness (*CS*(cor = 0.7) = 0.28). Node strength performed better (*CS*(cor = 0.7) = 0.75). Therefore, the closeness and betweenness indices were not reliable [16], whereas the strength was reliable.

**Fig 5:**
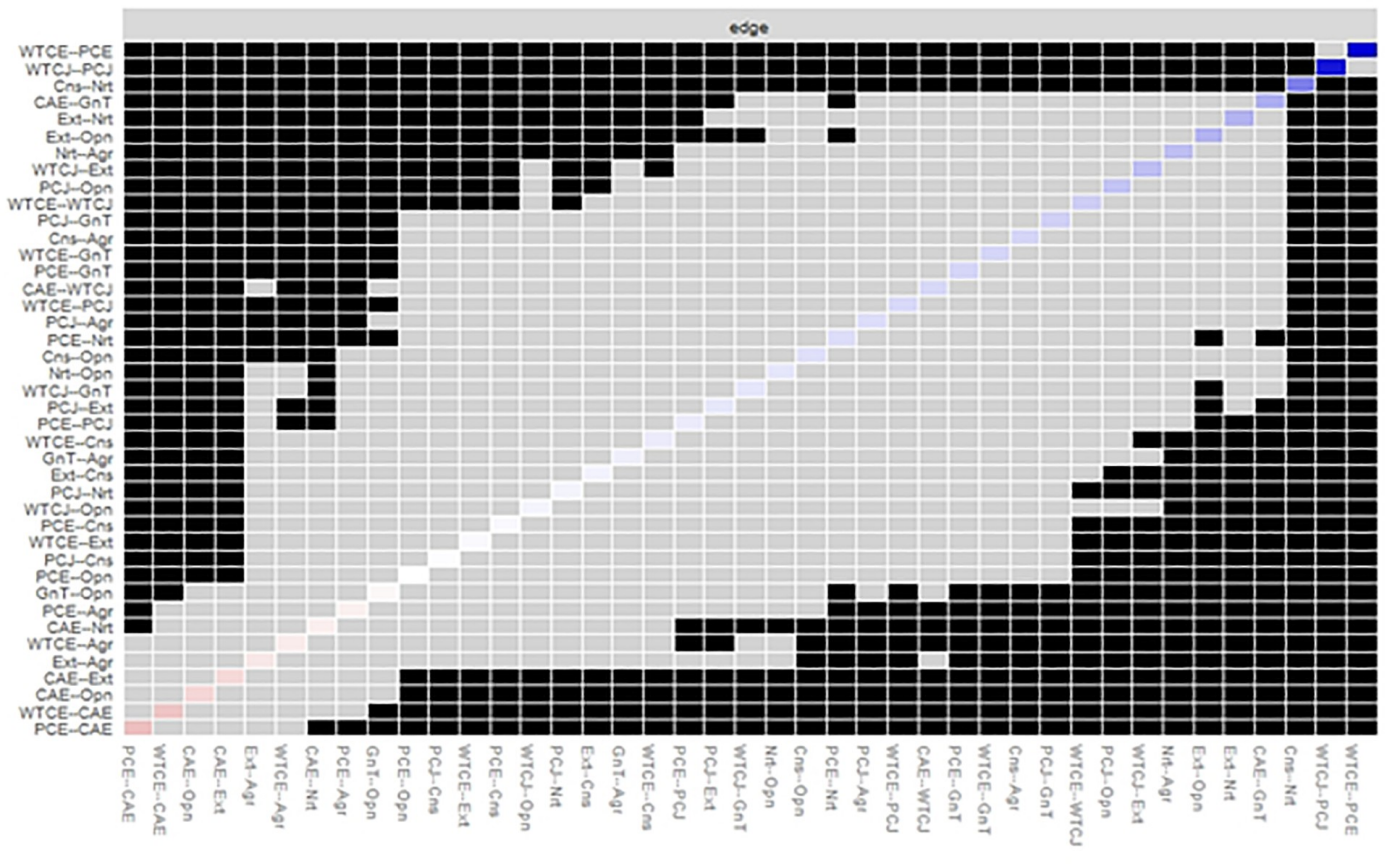
Bootstrapped differences (*α* = 0.05) between edge weights that were non-zero in the estimated network. Gray boxes indicate edges that do not differ significantly from one another. Black boxes indicate edges that differ significantly from one another. Colored boxes correspond to the colors of the edges in the estimated network (Not shown in this paper). The abbreviations are the same as Fig 1.

The *differenceTest* function was used to compare edge weights with the bootstrapped difference test. It uses non-parametric bootstrap results rather than case-dropping bootstrap results [16]. The *plot* function was used to plot the difference tests between all pairs of edges (Fig 5). The edges between perceived communication competence in English and the WTC in English, and between perceived communication competence in Japanese and the WTC in Japanese were different from the other edges, but similar to each other. The edge between conscientiousness and neuroticism was not the same as the others. The *plot* argument was used because the function plots bootstrapped CIs for edge weights. The *onlyNonZero* argument was used so that only the edges shown are non-zero in the estimated network; the order argument orders the edge weights from the most positive to the most negative edge weight in the sample network [16].

**Fig 6:**
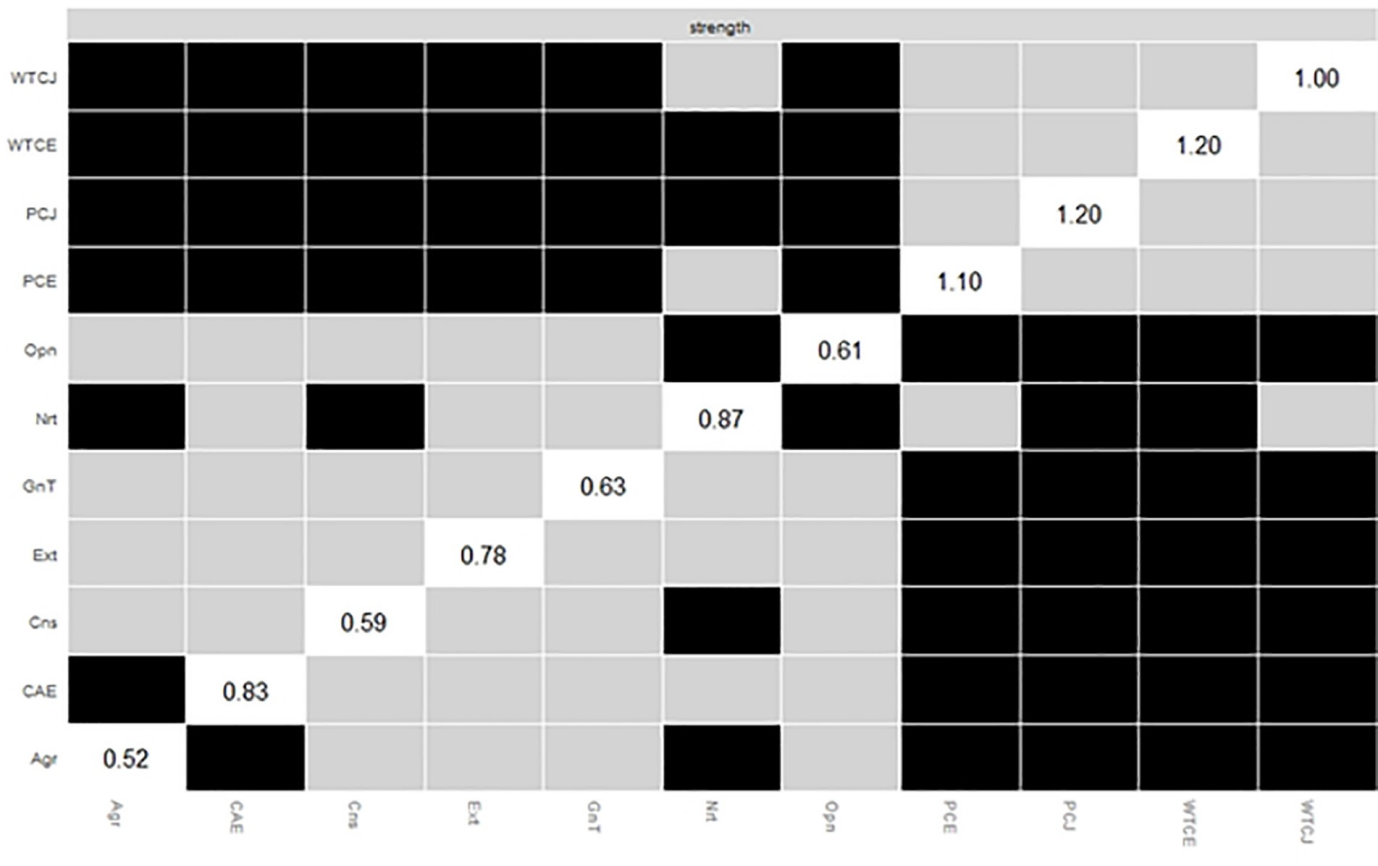
Bootstrapped differences (*α* = 0.05) between the node strengths of the factors. Gray boxes indicate nodes that do not differ significantly from one another. Black boxes indicate nodes that differ significantly from one another. White boxes in the centrality plot show the value of the node strength. The abbreviations are the same as Fig 1.

The *plot* function was also used to plot the differences in the centrality index node strength between all nodes (Fig 6). The perceived communication competence in English, WTC in English, perceived communication competence in Japanese, and WTC in Japanese had values over 1.0 and were not different from each other.

## Discussion

Before conducting the network analysis, the regression analysis of psychological factors on the WTC in English revealed that the effect of perceived communication competence in English was the strongest for the WTC in English, which is consistent with previous studies [22].

The LASSO regularization with EBIC model selection was used to examine the network structure of psychological factors and their effect on the WTC in English. The analysis revealed the interactions between the factors and their influence on the WTC in English. The edge between the perceived communication competence in English and the WTC in English was very strong, which is consistent with the regression analysis. Interestingly, the tendency was the same as the edge between the perceived communication competence in Japanese and the WTC in Japanese. The function of the perceived communication competence did not differ between the first and second languages. Similar to the regression analysis, general trust also had a positive connection with the WTC in English, while communication anxiety in English had a negative connection. The Big Five personality traits made a cluster and indirectly influenced the WTC in English; however, the effects were weak.

Node centrality strength showed that the WTC in English was central in the network. This means that the factor was the hub, and if it is removed, the network would become weaker. Additionally, the WTC in Japanese, perceived communication competence in Japanese, and perceived communication competence in English had scores around 1.0. These factors, which were related to language communication, worked as the central nodes and significantly influenced the WTC in English. We could see the result of closeness and betweenness as the centrality indices but their *CS coefficients* were low and not trustworthy. It was not ideal to interpret the network using betweenness and closeness in the present study. Furthermore, strength has been used a lot [16], and it is possible to interpret the network using only this index.

From these results, perceived communication competence in English simultaneously enhances the WTC in English and decreases communication anxiety. Furthermore, the WTC and perceived communication competence in Japanese consist of different clusters, which are mediated by communication anxiety in English, so if communication anxiety in English is activated, then the WTC in English is less activated, and the WTC in Japanese is more activated. MacIntyre and Charos [1] showed that if anxiety in a second language was activated, then the WTC was less activated, but they never showed that anxiety and WTC in a first language were activated simultaneously. This tendency suggests that if people feel anxiety in second language communication, they will depend on their first language for communication.

Bootstrapping methods were used to overcome replication concerns. The edges between the perceived communication competence in English and the WTC in English and between the perceived communication competence in Japanese and the WTC in Japanese are reliable because their bootstrapped CIs did not overlap with the bootstrapped CIs of any other edges. Similar to the thick connections of each edge in Fig 1, these edges had the strongest value in the network structure. Consistent with the previous study by Elahi et al. [22], perceived communicative competence in English had the greatest effect on WTC in English, even after controlling all the other edges.

The centrality indices of the estimated network models, based on data subsets, showed that the stability of closeness and betweenness decreased steeply, while that of the node strength improved. The *CS coefficients* of closeness and betweenness were not good, while node strength performed better. Therefore, the orders of node strength were interpretable in contrast to the orders of closeness and betweenness. Based on the results, we could only use the node strength to determine the central factor in the network structure.

Comparing edge weights using the bootstrapped difference test showed that the edges between the perceived communication competence in English and the willingness to communicate in English, and between the perceived communication competence in Japanese and the willingness to communicate in Japanese were significantly different from all other edge weights, but similar to each other. As shown in Fig 3, these edges were the strongest and significantly different in the network structure.

Comparing nodes using the bootstrapped difference test revealed that the node strength of the perceived communication competence in English, willingness to communicate in English, perceived communication competence in Japanese, and willingness to communicate in Japanese were high and not different from each other. Previous studies, to the best of the author’s knowledge, have never shown the strength as the centrality index of the communication variables and the central variables in the network structure. The present study showed the centrality of each component.

Psychology has shown that psychological factors influence human perception, affection, and behavior. However, the network structure of psychological factors and their effect on the WTC in a second language have not been reported. In this study, some factors positively influenced the WTC in English, while some discouraged it. Several factors related to language communication showed high centrality in the network structure. Furthermore, bootstrapping methods showed the trustworthiness of the estimated edge weights and centrality indices as well as the edges and node strengths that had significantly different effects than all the others.

Network analysis examined which psychological factor functioned as the center (hub) between the factors in relation to the WTC in English. If we could identify the centrality, we could determine the factor that is most essential for enhancing the WTC in English and the influence of each psychological factor [9]. Using this analysis, the effects can be seen visually; therefore, it is useful for interventions such as language education [9]. The results have implications for enhancing learners’ positive attitudes toward English communication.

As a result of the present study, the perceived communication competence in English was the central variable in the network. This finding suggests that if the competence in English is activated, the other variables such as the WTC in English are simultaneously activated directly and indirectly as well. From this point, in the EFL setting, through a positive feedback to learners’ communication behavior, teacher needs to promote their perceived competence in English to enhance the WTC in English and communication factors. Regression analysis could suggest that enhancing the perceived competence leads to a higher WTC, but the present study suggests that enhancing the perceived competence leads to a higher WTC and lower communication anxiety in English, and cyclically, increased WTC and decreased anxiety lead to perceived competence.

Not all participants had many opportunities to use English in their daily lives. Previous studies have shown the positive correlations between the WTC and frequency of communication in a second language [1, 6]. Therefore, the scale of willingness was useful to examine the relationships with other psychological factors. However, it is important to look at the actual foreign language communication behavior to make this study more convincing. A future study will examine the actual behavior to show the relationships with other psychological factors in the network.

General trust and personality could be at the different level from anxiety and confidence. According to MacIntyre and Charos [1], the Big Five personality traits directly and indirectly influenced second language WTC. Furthermore, Ito [5] showed that general trust was an important factor to predict WTC. Therefore, to examine the relationship between perceived communication competence, communication anxiety, and the WTC in English, these personality factors were important. However, other psychological constructs at the same level (e.g., value, motivation, different kinds of anxieties, and competences in other English skills) are also important, and the future study will examine these constructs in the network.

According to Yashima et al. [6], motivation to learn second language positively influenced the WTC in a second language via communication confidence (which consists of perceived communication competence and communication anxiety in English). In short, the communication confidence positively influenced the WTC in a second language directly, and it is considered as the crucial factor for WTC. Therefore, the present study focused on it. However, learning motivation and values to predict the WTC in a second language have been studied, and a future study should examine these factors.

MacIntyre et al. [2] showed the hieratical model of variables influencing WTC. According to the model, the layers of psychological factors differ from those of contextual factors. The present study focused on psychological factors only because the previous studies on psychological network focused on psychological factors such as depression. However, if the contextual factors are controlled in the network, the structure of network would change. A future study will include contextual factors in the network.

The inter-item correlations in the Big-Five scale, the “Ten-Item Personality Inventory” in the present study were lower compared to those in Oshio, Abe, and Cutrone [19]. These authors targeted university students (mean age = 19.20 years, *SD* = 1.50). In contrast, the present study focused on the social survey participants (mean age = 39.41 years, *SD* = 11.20). The lower inter-item correlations in the present study are due to different generations. A future study will examine the difference of the correlations among different ages.

It is possible to say that there is a reverse mechanism meaning that actual English communication increases competence and decreases anxiety. Furthermore, recent psychological network studies proposed a method that can examine causal relationships using longitudinal data [27, 28]. The present study is cross sectional, and caution is necessary when interpreting the causal relationship among variables. The future study will use the method that can examine the causal relationships using longitudinal data.

It is important to examine that each item or subfactor is included in psychological network analysis instead of mean scale scores for the WTC, anxiety, and competence. For example, Brandt, Sibley, and Osborne [29] used only items (e.g., “pNat” item or “pLab” item) and not scale scores (e.g., “symbolic belief component”). Network analysis at the item or subfactor level will see the central elements “within” anxiety and competence. Furthermore, clusters could be extracted for different contexts (“talking in dyads,” “small groups,” “large meetings,” and “audience” clusters) or different receivers (“strangers,” “acquaintances,” and “friends” clusters). The present study focused on the mean scale scores for all the variables, but the future study will conduct network analysis at the item or subfactor level.

## Conclusion

The present study showed the network structure of psychological factors and their effect on the WTC in a second language. The findings from our network analysis showed the interactions between factors and also determined the central factor. These findings have implications for enhancing learners’ positive attitudes toward second language communication.

## Supporting information

S1 Data Items of willingness to communicate in English. (DOCX)

S2 Data Items of general trust. (DOCX)

S1 Fig Network structure of psychological factors and the WTC in English after controlling for age and sex. (DOCX)

S2 Fig Centrality indices are shown as standardized z-scores after controlling for age and sex. (DOCX)

## References

[1] MacIntyre PD, Charos C. Personality, attitudes, and affect as predictors of second language communication. J Lang Soc Psychol. 1996;15: 3–26. doi: 10.1177/0261927X960151001

[2] MacIntyre PD, Clément R, Dörnyei Z, Noels KA. Conceptualizing willingness to communicate in a L2: A situational model of L2 confidence and affiliation. Mod Lang J. 1998;82: 545–562. doi: 10.1111/j.1540-4781.1998.tb05543.x

[3] Peng JE, Woodrow L. Willingness to communicate in English: A model in the Chinese EFL classroom context. Lang Learn. 2010;60: 834–876. doi: 10.1111/j.1467-9922.2010.00576.x

[4] Khajavy GH, MacIntyre P, Barabadi E. Role of the emotions and classroom environment in willingness to communicate: Applying doubly latent multilevel analysis in second language acquisition research. Stud Second Lang Acquis. 2018;40: 605–624. doi: 10.1017/S0272263117000304

[5] Ito T. The effect of general trust on communication in English. Paper presented at the 66th annual meeting of the Japanese Group Dynamics Association (Toyama, Japan). 2019.

[6] Yashima T, Nishide L, Shimizu K. The influence of attitudes and affect on willingness to communicate and second language communication. Lang Learn. 2004;54: 119–152. doi: 10.1111/j.1467-9922.2004.00250.x

[7] Yousef R, Jamil H, Razak N. Willingness to communicate in English: A study of Malaysian pre-service English teachers. Engl Lang Teach. 2013;6: 205–216. doi: 10.5539/elt.v6n9p205

[8] Fruchterman T, Reingold E. Graph drawing by force-directed placement. Softw Pract Exp. 1991;21: 1129–1164. doi: 10.1002/spe.4380211102

[9] Ito T. The influence of networks of general trust on willingness to communicate in English for Japanese people. Sci. Rep. 2020;10. doi: 10.1038/s41598-019-56089-4 33203991 PMC7672104

[10] Al-Murtadha MA. The relationships among self-reported and observed first language and second language willingness to communicate and academic achievement. Lang Cult Curric. 2020. 1–15. doi: 10.1080/07908318.2020.1727495

[11] Yamagishi T. The structure of trust: The evolutionary game of mind and society. Tokyo: University of Tokyo Press; 1998.

[12] Yamagishi T.Trust as a form of social intelligence. In: Cook KS, editor. Trust in Society. Russell Sage Foundation; 2001. pp. 121–147.

[13] Oz H. Big Five personality traits and willingness to communicate among foreign language learners in Turkey. SBP Journal. 2014;42: 1473–1482. doi: 10.2224/sbp.2014.42.9.1473

[14] MacIntyre PD, Babin PA, Clément R. Willingness to communicate: Antecedents and consequences. Commun. Q. 1999;47: 215–229. doi: 10.1080/01463379909370135

[15] Karadag S, Kaya SD. The effects of personality traits on willingness to communicate: A study on university students. Manas Journal of Social Studies. 2019;8: 397–410.

[16] Epskamp S. Network psychometrics. Unpublished doctoral dissertation, The University of Amsterdam. 2017. Available from: http://sachaepskamp.com/Dissertation#Cover_and_Table_of_Contents

[17] McCroskey JC. Reliability and validity of the willingness to communicate scale. Commun Q. 1992;40: 16–25. doi: 10.1080/01463379209369817

[18] Yamagishi T, Yamagishi M. Trust and commitment in the United States and Japan. Motiv Emot. 1994;18: 129–166. doi: 10.1007/BF02249397

[19] Oshio A, Abe S, Cutrone P. Development, reliability, and validity of the Japanese version of Ten Item Personality Inventory (TIPI-J). Jpn J Pers. 2012;21: 40–52. doi: 10.2132/personality.21.40

[20] Gosling SD, Rentfrow PJ, Swann WB. A very brief measure of the Big Five personality domains. J Res Pers. 2003;37: 504–528. doi: 10.1016/S0092-6566(03)00046-1

[21] Namikawa T, Tani I, Wakita T, Kumagai R, Nakane A, Noguchi H. Development of a short form of the Japanese Big-Five Scale, and a test of its reliability and validity. Jpn J Psychol. 2012;83: 91–99. doi: 10.4992/jjpsy.83.91 22834085

[22] Elahi Shirvan M, Khajavy GH, MacIntyre PD, Taherian T. A Meta-analysis of L2 Willingness to Communicate and Its Three High-Evidence Correlates. J Psycholinguist Res. 2019;48: 1241–1267. doi: 10.1007/s10936-019-09656-9 31342240

[23] Foygel R, Drton M. Extended Bayesian information criteria for Gaussian graphical models. Adv Neural Inf Process Syst. 2010;23: 2020–2028.

[24] Barber RF, Drton M. High-dimensional Ising model selection with Bayesian information criteria. Electron J Stat. 2015;9: 567–607. doi: 10.1214/15-EJS1012

[25] Epskamp S, Cramer A, Waldorp L, Schmittmann VD, Borsboom D. qgraph: Network visualizations of relationships in psychometric data. J Stat Softw. 2012;48: 1–18. doi: 10.18637/jss.v048.i04

[26] Friedman JH, Hastie T, Tibshirani R. glasso: Graphical lasso estimation of Gaussian graphical models [Computer software manual]. 2014. Available from: https://CRAN.R-project.org/package=glasso (R package version 1.8)

[27] Borsboom D, Cramer AOJ, Schmittmann VD, Epskamp S, Waldorp LJ. The small world of psychopathology. PLoS ONE. 2011. doi: 10.1371/journal.pone.0027407 22114671 PMC3219664

[28] Epskamp S, Fried EI. A tutorial on regularized partial correlation networks. Psychol Methods. 2018. doi: 10.1037/met0000167 29595293

[29] Brandt MJ, Sibley CG, Osborne D. What is central to political belief system networks? Pers Soc Psychol Bull. 2019;45: 1352–1364. doi: 10.1177/0146167218824354 30688553 PMC6676336

