# Supplementary material for: The influence of psychological network on the willingness to communicate in a second language
Source: PLoS One. 2021 Sep 17;16(9):e0256644. doi: 10.1371/journal.pone.0256644 (PMC8448313; doi:10.1371/journal.pone.0256644)
Supplement: S1 Data — (DOCX) [file pone.0256644.s001.docx]

S1 Data.

Talking with an acquaintance

Talking with a small group of acquaintances

Talking in a large meeting of acquaintances

Presenting a talk to a group of acquaintances

Talking with a stranger

Talking with a small group of strangers

Talking in a large meeting of strangers

Presenting a talk to a group of strangers

Talking with a friend

Talking with a small group of friends

Talking in a large meeting of friends

Presenting a talk to a group of friends
