# Supplementary material for: The influence of psychological network on the willingness to communicate in a second language
Source: PLoS One. 2021 Sep 17;16(9):e0256644. doi: 10.1371/journal.pone.0256644 (PMC8448313; doi:10.1371/journal.pone.0256644)
Supplement: S2 Data — (DOCX) [file pone.0256644.s002.docx]

S2 Data.

Most people are trustworthy

Most people will respond in kind when they are trusted by others

Most people are trustful of others

Most people are basically honest

I am trustful

Most people are basically good and kind
