# Supplementary figures and images for: The influence of psychological network on the willingness to communicate in a second language
Source: PLoS One. 2021 Sep 17;16(9):e0256644. doi: 10.1371/journal.pone.0256644 (PMC8448313; doi:10.1371/journal.pone.0256644)

S1 Fig.


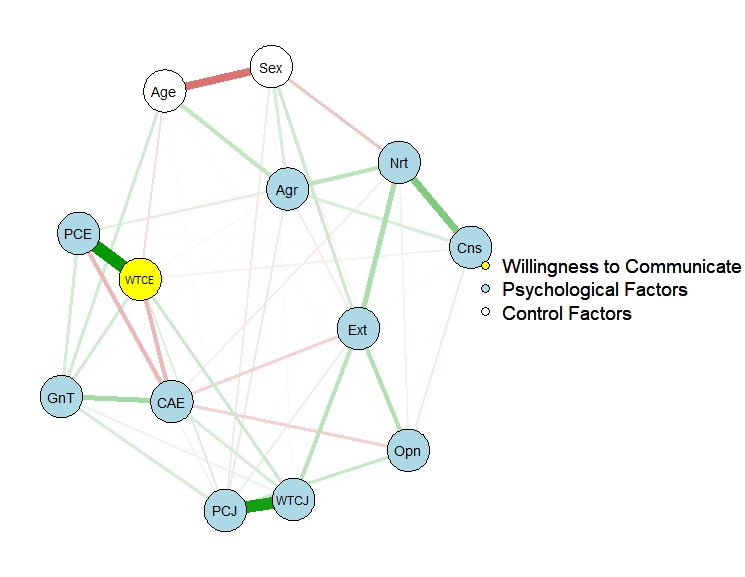

Supplement: S1 Fig — (DOCX) [file pone.0256644.s003.docx]

S2 Fig.


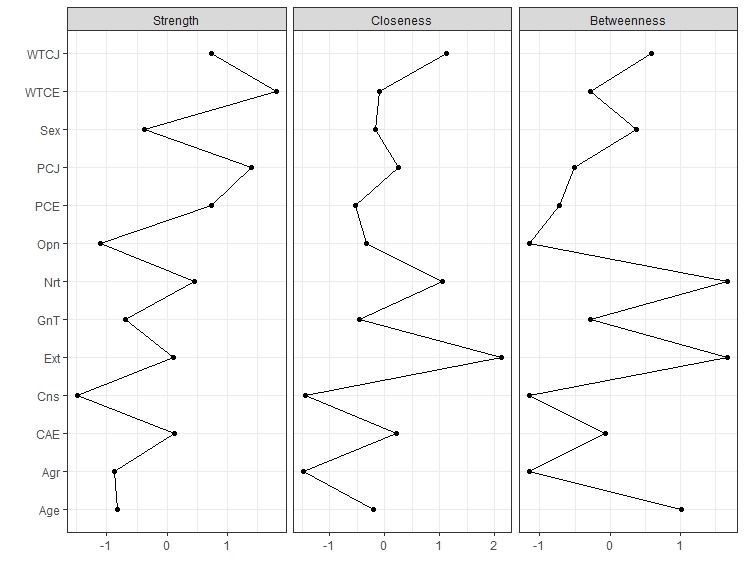

Supplement: S2 Fig — (DOCX) [file pone.0256644.s004.docx]
